# An Innovative Solution Based on TSCA-ViT for Osteosarcoma Diagnosis in Resource-Limited Settings

**DOI:** 10.3390/biomedicines11102740

**Published:** 2023-10-10

**Authors:** Zengxiao He, Jun Liu, Fangfang Gou, Jia Wu

**Affiliations:** 1 School of Computer Science and Engineering, Central South University, Changsha 410083, China; 8204200201@csu.edu.cn; 2 The Second People’s Hospital of Huaihua, Huaihua 418000, China; 3 State Key Laboratory of Public Big Data, College of Computer Science and Technology, Guizhou University, Guiyang 550025, China; gff8221@163.com; 4 Research Center for Artificial Intelligence, Monash University, Melbourne, Clayton, VIC 3800, Australia

**Keywords:** cell nucleus segmentation, osteosarcoma, machine learning, image noise reduction, computational efficiency, pathology slide images, resource-limited settings, twin-self and cross-attention vision transformer (TSCA-ViT)

## Abstract

Identifying and managing osteosarcoma pose significant challenges, especially in resource-constrained developing nations. Advanced diagnostic methods involve isolating the nucleus from cancer cells for comprehensive analysis. However, two main challenges persist: mitigating image noise during the capture and transmission of cellular sections, and providing an efficient, accurate, and cost-effective solution for cell nucleus segmentation. To tackle these issues, we introduce the Twin-Self and Cross-Attention Vision Transformer (TSCA-ViT). This pioneering AI-based system employs a directed filtering algorithm for noise reduction and features an innovative transformer architecture with a twin attention mechanism for effective segmentation. The model also incorporates cross-attention-enabled skip connections to augment spatial information. We evaluated our method on a dataset of 1000 osteosarcoma pathology slide images from the Second People’s Hospital of Huaihua, achieving a remarkable average precision of 97.7%. This performance surpasses traditional methodologies. Furthermore, TSCA-ViT offers enhanced computational efficiency owing to its fewer parameters, which results in reduced time and equipment costs. These findings underscore the superior efficacy and efficiency of TSCA-ViT, offering a promising approach for addressing the ongoing challenges in osteosarcoma diagnosis and treatment, particularly in settings with limited resources.

## 1. Introduction

The incidence of cancer is a significant global health challenge, featuring 19.3 million identified instances and 10 million fatalities linked to cancer reported in 2020 [1]. Cancer significantly contributes to mortality rates globally and poses a considerable challenge to the enhancement of life expectancy [2]. Moreover, the financial burden associated with cancer identification and management is substantial. For example, China commits more than 220 billion yuan annually to combat malignant tumors. Within the spectrum of cancers, bone cancer stands out as one of the most common primary malignant bone neoplasms. Pathologically defined as a sarcoma, this type of cancer is characterized by its production of a bone-like matrix or osteoid, which can be directly observed under a microscope [3,4]. It ranks second in the incidence of primary malignant tumors [5,6]. Adolescents are the most affected population, followed by elderly people over 60 years old [7]. In developing countries, limited medical resources often contribute to higher mortality rates and late-stage diagnoses for bone cancer patients compared to those in developed nations. Regrettably, the five-year survival rate for late-stage osseous neoplasm stands at a modest 20% [8].

As computer technology constantly evolves and progresses, artificial intelligence represents the forefront of contemporary computer applications. It was initially proposed by McCulloch and Pitts, who introduced a mathematical model called “artificial neuron.” As deep learning technology has surfaced [9,10], artificial intelligence has made remarkable advancements in areas like image recognition and natural language processing [11]. Currently, artificial intelligence medical assistance systems are widely implemented in the healthcare domain [12]. In the treatment of COVID-19, artificial intelligence medical assistance systems use deep learning to fight against the virus, analyze large amounts of clinical data and medical images to accelerate the development of drugs, and also expedite the identification and treatment process of COVID-19 [13,14]. In computer-aided medical image segmentation, artificial intelligence greatly improves the efficiency of medical staff by assisting in the processing of numerous clinical images [15,16].

While these innovations have been employed in certain medically progressive areas and have addressed numerous issues [17,18], applying them to the healthcare systems of developing countries presents challenges due to the following reasons:

Firstly, the cost of bone sarcoma pathology section nuclear detection is high, with high instrument and equipment consumables [19]. In most centers in developing countries, auxiliary technologies are not used to assist diagnosis because funds, resources, and materials are unavailable [20], and patients cannot afford the additional cost of these technologies [21]. Therefore, the actual cost of auxiliary diagnosis needs to be carefully considered [22]. Secondly, developing countries generally face the problem of insufficient per capita diagnosis and medical resources, and imbalanced allocation of healthcare resources [16,23,24]. China is a typical developing country with significant differences regarding the distribution of healthcare resources between urban and rural regions, including medical expenses, the number of medical devices, available beds, and personnel. Over 80% of healthcare resources are focused in advanced areas, which are home to a mere 10% of the population [25]. Therefore, solving the diagnosis problem of bone sarcoma pathology has a significant role in helping to tackle the imbalanced allocation of healthcare resources among developing nations [26,27,28].

Moreover, due to limited economic conditions in developing countries, equipment is generally outdated [28,29], and a large amount of complex noise interference can easily occur during pathological testing, seriously affecting the final detection efficiency and accuracy [30]. Additionally, developing countries also have a certain tense doctor–patient relationship [31]. Owing to the constraints in healthcare resources [32], primary hospitals in developing nations, and even some provincial and municipal hospitals, are still unfamiliar with bone sarcoma tissue sections [33]. Inexperienced doctors are likely to misdiagnose this disease, which causes irreversible consequences to patients and easily worsens the already tense doctor–patient relationship, leading to a significant negative impact on overall social welfare [34]. Therefore, we must improve the accuracy of AI medical assistance design and find an equilibrium between swiftness and precision [11].

In this article, we address critical challenges associated with the imaging and transmission of osseous neoplasm pathological section images. Specifically, noise data generated during these processes can severely disrupt both manual diagnoses by medical professionals and automated segmentation by intelligent models. To tackle this, we implement a directed filtering technique designed to remove noise, thereby increasing the accuracy of automated segmentation. Taking into account the need for computational efficiency in real-world applications, we introduce an innovative transformer framework equipped with an ultraefficient dual-attention mechanism. This approach is inspired by the U-Net architecture but excludes convolutional layers, significantly reducing computational complexity. Furthermore, we enhance the model’s localization capabilities by redesigning the skip connection pathways using cross-attention components. These collective improvements aim to advance the field of medical image analysis by providing more precise and efficient tools for healthcare professionals:

(1) We deployed a novel directed filtering algorithm to improve the quality of pathological image data, which is often affected by various factors like device noise and accidental errors in the sample preparation process. By using the grayscale image of the original picture as guidance, our approach effectively eradicates noise, thereby preserving the image’s texture and offering excellent edge detection. This enhancement in data quality, in turn, significantly boosts the performance of our deep learning model.
(2) Our work introduces an innovative Transformer framework equipped with an efficient twin attention mechanism for sophisticated modeling and segmentation. Utilizing patch-embedding modules, our network attains overlapping patch labels, which are then encoded through an encoder module to gain hierarchical, multiscale representations. Further, we optimized the attention mechanism for efficiency, substantially reducing computational complexity while ensuring high representativeness.
(3) We devised skip connection paths integrated with cross-attention modules to furnish each decoder with spatial information. Coupled with the efficient attention mechanism, this strategy strengthens the model’s localization ability, thereby improving the final model’s precision while maintaining model reusability.
(4) We experimented with a dataset of 1000 pathological images from the Second People’s Hospital of Huaihua. Our experimental findings showcase the advantage of our method in comparison to other convolutional and non-convolutional segmentation networks in segmenting the nuclei of osseous neoplasm cells in pathological sections.

The rest of this paper unfolds in the following manner: Section 2 describes related work, introduces some methods for denoising images and mainstream methods and ideas for medical image segmentation in recent years, and explains the motivation behind our proposed design. Section 3 introduces the system model for implementing our solution. Section 4 describes the dataset, evaluation metrics, and in the experimental results section, we illustrate the efficacy of our methodology. In the end, we conduct a comprehensive analysis of our experimental findings, highlight areas for potential improvement in our work, and propose directions for future research.

## 2. Related Work

The development of AI medical assistance in diagnosis relies heavily on a large number of clinical practices and research [35]. Using these intelligent assistance systems to analyze pathological sections can greatly improve the efficiency and quality of doctors’ work, saving a lot of valuable time and energy resources for the medical system. Therefore, we hope to achieve high accuracy in segmentation results with minimal time and computational device costs by quickly and effectively filtering out noisy interference [36] that appears in pathological sections [26,37,38].

Denoising of pathological sections is one of the important tasks in the diagnosis of AI-assisted medical systems [20]. Noise in images often appears as isolated pixels or blocks of pixels that strongly affect the visual effect and are unrelated to the pathological images we are studying, which makes the pathological sections unclear [39]. Various sources of disturbances can compromise the quality of captured images. These may arise from intrinsic factors, such as the properties of the sensor materials, or from external conditions affecting the working environment. Additionally, imperfections in transmission media and devices can introduce noise during the image signal transmission process. This is particularly relevant for developing countries, where equipment-related noise can significantly interfere with medical diagnoses [40].

As the field of deep learning continues to evolve, its applications have broadened to include sophisticated denoising algorithms across multiple domains. One notable example is the Total Variation denoising algorithm, which employs gradient descent techniques to enhance image quality. However, this algorithm presents challenges in terms of hardware prerequisites and computational complexity, requiring advanced equipment and resulting in high time consumption [41]. Conventional denoising techniques like mean filtering and Gaussian filtering employ isotropic filters. These filters take a uniform approach to both noise and edge information. While they are effective in smoothing out noise, they have the drawback of also eliminating crucial details such as image edges, textures, and other fine features [42]. Therefore, we choose the Directed Filtering technique to help us achieve denoising of pathological images. Directed filtering is an image filtering technique that filters the starting image (input) via a guiding map, ensuring the resulting output image closely resembles the original image while maintaining texture similarity with the guidance map [43]. Compared with other methods, the directed filtering we used on our pathological slide dataset can not only achieve edge smoothing of bilateral filtering, but also perform well in detecting edge attachments, while having a smaller time complexity [6,44,45].

Medical semantic segmentation holds vital significance in the segmentation of pathological images. Efficient and accurate image segmentation algorithms are important for assisting medical diagnosis and treatment. Convolutional neural networks (CNN) have emerged as prominent players in this field [46]. With the rise of classic convolutional models such as U-Net [47] and FCN [48], their applications in pathological slides have also been expanding.

Recent work, such as our prior study on the Transformer-based solution for osteosarcoma tissue (OstT), and TBNet, which combines Transformer and U-Net architectures, have shown the promise of machine learning techniques in this context, yet challenges remain [22].

The DU-Net algorithm [49], based on U-Net [47], has shown good performance in segmenting gastric cancer pathological slides. It divides the dataset into small pieces and uses the model for segmentation, followed by postprocessing utilizing a fully connected Conditional Random Field (CRF), for enhancing the segmentation outcomes. Nonetheless, in spite of the commendable performance of convolutional neural networks in semantic segmentation of pathological slides, they still have limitations in capturing shape and structural information and lack efficiency. Furthermore, there are significant segmentation differences in size and shape among different pathological slide images [50]. To address the limitations of convolutions, the Visual Transformer (ViT) [51] was proposed, which relies solely on the multi-head self-attention mechanism. The CASTformer [52] proposed a hybrid network structure that combines CNN and Transformer; incorporating hierarchical pyramid structures enables the acquisition of ample global spatial information and local context details at various scales. The results showed that CASTformer is a powerful initiation point for subsequent medical imaging analysis activities.

In order to address the weak representation limitations of Transformers, TransUNet [53] employs the convolutional neural network’s feature maps as input sequences for Transformer layers, and integrates convolutional kernel attention for capturing both global and intricate local contexts, attaining outstanding results in medical image segmentation; nevertheless, owing to the extensive quantity of parameters, low computational efficiency, and heavy reliance on the CNN backbone network, TransUNet has certain limitations.

To tackle the intricacy of Transformer architectures, contemporary designs primarily concentrate on computing channel attention [24], imposing certain restrictions on locally performed token attention mechanisms [54], or defining scaling factors to reduce spatial dimensions [55]. Although these methods have some reference value in reducing model complexity, they only partially capture global context. Swin-Unet [56], designed for medical image semantic segmentation, uses two consecutive transformer blocks to retrieve context from adjacent windows. While it strengthens feature representation from multiple dimensions, there is still ample room for development in capturing spatial context during the process.

To overcome the limitations of traditional convolutional models and address the challenges posed by the recently developed Vision Transformer (ViT) in capturing contextual information, we introduce an innovative Transformer framework guided by dual attention mechanisms for medical semantic segmentation in computer vision [57]. Experimental outcomes reveal that TSCA-ViT attains superior segmentation precision and consistency while preserving minimal computational expenses and outperforms various convolutional network methods on the same dataset without the need for weight loading.

## 3. System Model

**Figure 1:**
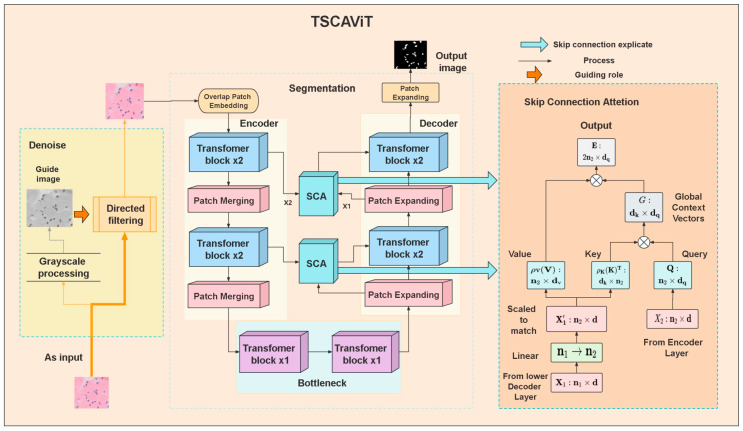
Overall TSCA-ViT architecture.

Owing to the large amount of data in pathological slices of osseous neoplasm cells [58], semantic segmentation of pathological slices can assist doctors in diagnosing and treating patients, which is a huge challenge for developing countries. Therefore, with the development of AI-assisted medical systems, we hope to help medical professionals segment pathological slices using the intelligent model we have designed, reducing their workload and saving valuable clinical diagnostic time, while providing strong data evidence for clinical diagnosis. The system model we propose is shown in Figure 1. First, our original images are batch-processed using directed filtering to quickly reduce noise, remove a large amount of clutter noise that interferes with image segmentation, and improve quantitative measurement of the image. Then, we convert the denoised images into sequence embeddings and segment them into nonoverlapping patches. We use a Transformer [55] comprising an encoder and decoder to carry out the segmentation procedure, and restructure the skip connection route by incorporating cross-attention components. In the final step, we utilize a linear projection layer on the extracted features to produce pixelwise partitioning forecasts. This study is divided into two primary sections: the first focuses on noise reduction in the initial visual imagery, while the second tackles the development of a linguistic segmentation model specifically designed for the nuclei of osseous neoplasm cells. Table 1 provides a comprehensive overview of the equations detailed within this paper’s figures.

### 3.1. Image Denoising

Image noise constitutes an unavoidable form of extraneous interference that occurs during the imaging of osseous neoplasm pathological sections. Additional equipment-related interference also arises during the conversion and transmission of these slice images. Such interferences introduce a substantial amount of noise, primarily in the form of Gaussian and salt-and-pepper noise, into the image analysis process. This notably compromises the accuracy of the model’s segmentation capabilities, thereby affecting overall performance.

Many traditional denoising methods rely on isotropic filtering, which uniformly treats noise and edge information. While effective in reducing noise, these methods often compromise the finer details, including edges and textures, in the image. In contrast, directed filtering uses a reference image to guide the processing of the original image. The resulting output retains the general appearance of the initial image but incorporates texture elements that are consistent with the reference image. Directed filtering not only excels in smoothing edges, akin to bilateral filtering, but also performs admirably in detecting edges in adjacent regions.

The filter’s mathematical formula can be described as:

(1) $$p_{i}=\sum_{j\in w_{i}}M_{ij}\left(K\right)\cdot q_{j}$$

where K is the guiding image, $q_{j}$ is the input filtered image, $p_{i}$ is the filtered output image. Generally, we choose the grayscale image of the original color image as the guiding image K, or perform some edge-preserving filtering operations on the grayscale image to use it as the guiding image. $M_{ij}$ is the weight value used in the weighted averaging operation determined by the guiding image *K*. Its expression is as follows:

(2) $$M_{ij}\left(K\right)=\frac{1}{|\omega {|}^{2}}\sum_{k\left(i,j\right)\epsilon \omega_{k}}\left(1+\frac{\left(K_{i}-\mu_{k}\right)\left(K_{j}-\mu_{k}\right)}{\sigma_{k}^{2}+\epsilon }\right)$$

In the formula, $\mu_{k}$ is the mean value of the pixel points within the $\omega_{k}$ window, $K_{i}$ and $K_{j}$ refer to the values of two adjacent pixels, $\omega_{k}$ signifies the quantity of pixels encompassed within the window, $\sigma_{k}^{2}$ represents the square difference of the pixel points within the $\omega_{k}$ window, and ϵ is a penalty value. The adaptive weight can be analyzed based on the formula above: when $K_{i}$ and $K_{j}$ are on opposite sides of the boundary, ($K_{i}$ − $\mu_{k}$) and ($K_{j}$ − $\mu_{k}$) have opposite signs; otherwise, they have the same sign. The weight value when they have opposite signs is much smaller than the weight value when they have the same sign. Therefore, the pixels in the flat region are given a larger weight, resulting in a more pronounced smoothing effect. The pixels on both sides of the boundary are given a smaller weight, resulting in a weaker smoothing effect, but can help maintain the edge.

The penalty parameter ϵ significantly influences the filtering outcome. With a smaller *ϵ* value, the filtering behaves as previously mentioned. Conversely, when *ϵ* is larger, the weight calculation formula approximates a mean filter, leading to a more pronounced smoothing effect.

(3) $$p_{i}=q_{i}-n_{i}$$

(4) $$p_{i}=aK_{i}+b$$

As shown in these two formulas, assuming that $p_{i}$ is the linear transformation of I in the window with pixel K as the center, by guiding through the K map, the generated image texture is similar to that of K, which helps us retain the texture features of pathological slices and increase the accuracy of model segmentation. However, since we cannot know the specific value of each $n_{i}$, we try to calculate the desired image through the guidance map. That is to say, we can only know which ones are edges and which ones are regions by using the linear guidance correlation between the guidance map K and the filtering output $p_{i}$.

From the perspective of the formula of linear filtering:

(5) $$p_{i}=a_{k}K_{i}+b_{k},\forall i\in \omega_{k}$$

Among them, $a_{k}$ and $b_{k}$ are considered constant linear coefficients in the $\omega_{k}$ window. In order to determine the linear coefficients $a_{k}$ and $b_{k}$, we need constraints from the filtered input q. We model the output p by subtracting some unwanted components n from the input q, such as noise/texture. The expressions are as follows:

(6) $$p_{i}=q_{i}-n_{i}$$

(7) $$a_{k}=\frac{\frac{1}{\left|\omega \right|}\sum_{i\in \omega_{k}}K_{i}p_{i}-\mu_{k}{\bar{q}}_{k}}{\sigma_{k}^{2}+\epsilon }$$

(8) $$b_{k}={\bar{q}}_{k}-a_{k}\mu_{k}$$

where $\mu_{k}$ is the mean value, and $\sigma_{k}^{2}$ is the variance of I in the window $\omega_{k}$, $\left|\omega \right|$ is the number of pixels in the window $\omega_{k}$. In addition, ${\bar{q}}_{k}$ is the average value of the input.

When K = q, it simplifies to:

(9) $$a_{k}=\frac{\sigma_{k}^{2}}{\sigma_{k}^{2}+\epsilon }$$

(10) $$b_{k}=\left(1-a_{k}\right)\mu_{k}$$

If ϵ=0, it is obvious that a=1 and b = 0 are the solutions that minimize E(a,b). From the above equation, it can be seen that in this case, the filter has no effect and simply outputs the input as it is.

If ϵ>0 and the pixel intensity changes are small in the region (low variance), i.e., the image I remains essentially constant in the window $\omega_{k}$, then $\sigma_{k}^{2}\;$<< ϵ, which implies that ak ≈ 0 and bk ≈ μk, resulting in a weighted mean filter. In high variance regions, where the image I changes significantly in the window $\omega_{k}$, $\sigma_{k}^{2}\;$>> ϵ, which leads to ak≈1 and bk≈0, resulting in weak filtering that preserves edges. With a fixed window size, as ϵ increases, the filtering effect becomes more pronounced.

Additionally, during the calculation of linear coefficients for each window, a pixel may be part of several windows, implying that multiple linear functions describe each pixel. Consequently, as stated earlier, to determine the output value for a specific point, we simply need to compute the average of all linear functions encompassing that point (local linear model), as illustrated below (Algorithm 1):

(11) $$q_{i}=\frac{1}{\left|w\right|}\sum_{k:i\in w_{k}}\left(a_{k}K_{i}+b_{k}\right)=\bar{a_{i}}K_{i}+\bar{b_{i}}$$

**Algorithm 1**: Guided Filtering algorithm**Input:** filtering input image q, guidance image K, radius r, regularization ϵ **Output:** filtering output p
$1:{mean}_{K}=f_{mean}(K)$ ${mean}_{q}=f_{mean}(q)$ ${\;corr}_{K}=f_{mean}(K.\times K)$
${corr}_{Kq}=f_{mean}(K.\times q)$ $2:{var}_{K}={corr}_{K}-{mean}_{K}\times {mean}_{K}$ ${cov}_{Kq}={corr}_{Kq}-{mean}_{K}\times {mean}_{q}$ $3:a={cov}_{K_{Kq}}./({var}_{K}+\epsilon )$ $b={mean}_{q}-a.\times {mean}_{K}$ $4:{mean}_{a}=f_{mean}\left(a\right)$ ${mean}_{b}=f_{mean}\left(b\right)$ $5:p={mean}_{a}\cdot \times K+{mean}_{b}$ End for; End for

Above is a linear regression equation, where $f_{mean}$ is the function for computing the mean, q and K are arrays, ${var}_{K}$ is the variance of K, ${cov}_{Kq}$ is the covariance between K and q, a and b are the regression coefficients, ${mean}_{a}$ and ${mean}_{b}$ are the mean values of a and b, and p is the predicted result.

The computational intricacy relies on the execution and dimensions of the array structures q and K. If $f_{mean}$ is implemented as a simple mean calculation, the time complexity for each mean calculation is O(N), where N represents the cumulative sum of components within the array. The computational time complexity for determining variance and covariance is also O(N). The computational duration required for determining a and b is O(1), while the time complexity for computing ${mean}_{a}$ and ${mean}_{b}$ is O(N). The time complexity for prediction computation is O(N).

After applying directed filtering for denoising, we successfully eliminated the majority of noise present in the original sliced images. Subsequent bilateral filtering enhanced edge smoothing and clarity, while also preserving maximal texture information in the images. These preprocessing steps led to a substantial increase in the segmentation accuracy of the denoised images.

### 3.2. Image Analysis and Prediction

**Figure 2:**
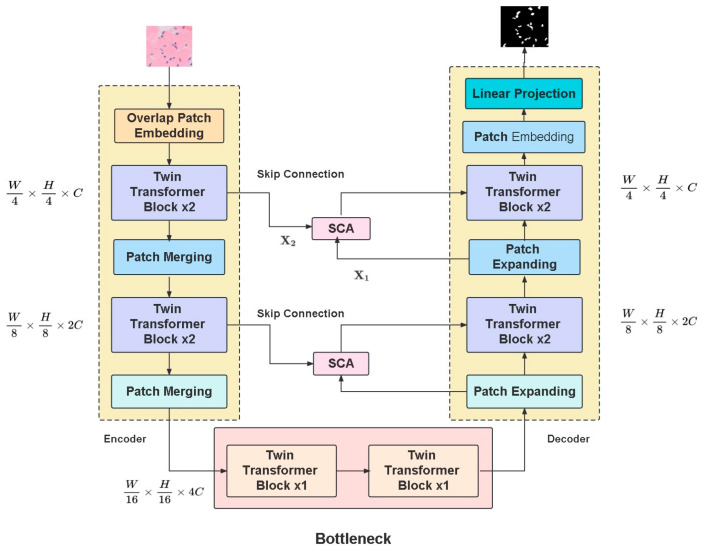
Schematic diagram of segmentation.

The TSCA-ViT network segmentation model for osseous neoplasm pathological sections is a purely Transformer-based structure devoid of convolution, resembling a U-Net-like layer-based architecture, as illustrated in Figure 2. Upon receiving an input image, the TSCA-ViT model reduces its resolution by a factor of 4 using the patch embedding module, resulting in overlapping patch labels. The tokenized image undergoes processing by the encoder module, which is composed of a series of three encoder blocks, each featuring a pair of successive Dual Transformer strata along with a singular Patch Merging layer. The patch merging operation combines 2×2 patch tokens, simultaneously reducing the spatial size, and by increasing the channel size twofold, the network has the capacity to depict features of multiple scales in a hierarchical manner. During the decoding phase, token dimensions undergo a double expansion. Subsequently, output from each patch expansion layer is integrated alongside the characteristics relayed from the matching encoding layer via Skip Connection Attention (SCA). Lastly, the generated features are subjected to two successive Dual Transformer strata and one linear projection layer generate the concluding partitioning representation.

Broadly speaking, integrating spatial and channel attention can significantly enhance the model’s ability to grasp a greater range of contextual features. Consequently, we devised a Dual Transformer unit that combines inverse (channel-based) attention and effective (space-oriented) attention.

The efficient attention and add and norm expressions are provided below:

(12) $$E_{block}\left(X,Q_{1},K_{1},V_{1}\right)=E\left(Q_{1},K_{1},V_{1}\right)+X$$

(13) $${MLP}_{1}\left(E_{block}\right)=\mathrm{MLP}\left(LN\left(E_{block}\right)\right)$$

E(·) refers to efficient attention, $E_{block}$ refers to efficient attention block, and $Q_{1},K_{1},V_{1}$ are the query, key, and value calculated based on the input feature X, and MLP represents the mixed FFN feed-forward network.

The expression is as follows:

(14) $$\mathrm{MLP}\left(X\right)=FC\left(\mathrm{GELU}\left(DW-\mathrm{Conv}\left(FC\left(X\right)\right)\right)\right)$$

Here, FC denotes a fully connected layer, while GELU signifies GELU activation, and DW−Conv is a depthwise convolution. The expression for the transpose attention block and add and norm for channel attention are as follows:

(15) $$T_{block}\left(E_{block},Q_{2},K_{2},V_{2}\right)=T\left({MLP}_{1}\left(E_{block}\right)+E_{block}\right)+{\mathrm{MLP}}_{1}\left(E_{block}\right)$$

(16) $${\mathrm{MLP}}_{2}\left(T_{block}\right)=\mathrm{MLP}\left(LN\left(T_{block}\right)\right)$$

where T(·) refers to transpose attention, $T_{block}$ represents transpose attention block, $E_{block}$ refers to efficient attention block, and MLP represents Mix−FFN feed-forward network.

The final expression of the dual attention block is:

(17) $$DualAttention\left(T_{block}\right)={\mathrm{MLP}}_{2}\left(T_{block}\right)+T_{block}$$

**Figure 3:**
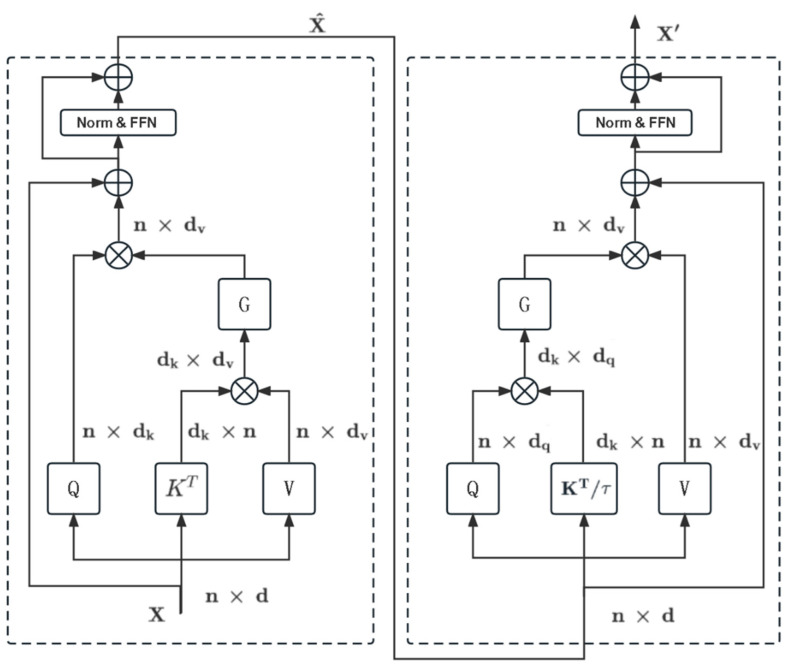
Effective Twin Attention Module.

The architecture is illustrated in Figure 3.

When it comes to the Effective Dual Attention Module, it includes an effective attention block, followed by “Norm&FFN”, and a channel attention block, subsequently utilizing “Norm&FFN” to conduct both spatial and channel attention.

In the encoder, the input resolution consists of a C-dimensional tokenized input block with a resolution of H4×W4, which is then fed into each block comprising two sequential Dual Transformers and a patch merging layer. After this calculation, the input resolution becomes 2C dimensions with a resolution of H8×W8. During the patch merging process, the spatial dimension is reduced by merging 2×2 patch tokens, concurrently doubling the channel size, and the network is empowered to acquire multiscale representations in a layered manner. Finally, it is sent to the bottleneck, where its dimension resolution becomes 4C dimensions with a resolution of H16×W16. In addition, in the two Dual Transformer layers, skip connection input $X_{2}$ is designed.

Within the patch merging layer, the input patch is segmented into four sections and combined by a patch merging layer, subsequently decreasing the feature resolution by half (down sampling 2X) and increases the dimension by four times (continuous operations lead to a 4X increase in feature dimension). Therefore, a linear layer is applied to the combined features to strengthen the feature dimension, effectively doubling its initial size. For the bottleneck, we only use two consecutive Transformer layers to build the bottleneck model due to the excessive depth of the Transformer model to train an optimal solution. This allows for the acquisition of more profound feature representations, while the bottleneck model preserves the feature size and resolution. By selecting two consecutive Dual Transformers, we achieve the goal of feature dimension reduction, saving computational resources, and increasing the nonlinear expression capacity of our model.

To match the encoding process, we designed a decoder that uses the Dual Transformer block in a symmetrical way. This block doubles the size of each token and upscales the extracted features through a patch expansion layer. The patch expansion layer modifies the adjacent-dimension feature maps, transforming them into higher-resolution feature maps through 2X upsampling while concurrently reducing the feature dimension by half compared to its initial size. The outcome of every patch expansion layer is integrated with the corresponding features forwarded from the parallel encoder layers through skip connections using the SCA module. The produced features are directed through two successive Dual Transformer layers, ultimately emitting output via a linear projection layer.

For the initial convolutional layer expansion within the patch expansion layer, input features (W16×H16×4C) undergo a linear layer transformation before upsampling to double the feature dimension to the original dimension (W16×H16×4C) two times. Afterward, a reshaping operation is conducted to double the input feature resolution, while simultaneously decreasing the feature dimension to one-fourth of its initial size (W16×H16×4C→W4×H4×C).

As for the transposed attention, we use cross-covariance attention, also known as transposed attention, employing channel attention as a mechanism. This approach solely employs transposed attention for handling larger input sizes. We propose a new transposed attention mechanism to comprehensively capture the entire channel dimension, which is formulated as follows:

(18) $$T\left(Q,K,V\right)=VC_{T}\left(K,Q\right),C_{T}\left(K,Q\right)=\mathrm{Softmax}\left(\frac{K^{T}Q}{\tau }\right)$$

T(·) denotes transposed attention, with Q, K, and V representing keys, queries, and values, respectively. In this instance, key and query matrices are transposed, resulting in attention weights based on cross-covariance matrices. $C_{T}$ symbolizes the context vector for transposed attention. By incorporating a temperature parameter τ, an L2 normalization is conducted on both queries and keys, offsetting scaling effects to enhance training stability while marginally diminishing the module’s representational capacity.

The spatial complexity of transposed attention is $O(hN^{2}+Nd)$, while that of self-attention is $O(d^{2}/h+Nd)$. Self-attention exhibits a quadratic relationship concerning the number of tokens N; while transposed attention exhibits quadratic dependence on the embedding dimension d, it is typically smaller than N, particularly for images of larger size.

An efficient attention module incorporating the original Transformer’s computation formula is presented below:

(19) $$S\left(Q,K,V\right)=\mathrm{SoftMax}\left(\frac{QK^{T}}{\sqrt{d}}+B\right)V$$

The computational complexity exhibits a quadratic relationship concerning the input tokens, which substantially restricts the suitability of this architecture for high-resolution images. Consequently, we employ optimized computational approaches:

(20) $$E\left(Q,K,V\right)=\rho_{q}\left(Q\right)(\rho_{k}(K{)}^{T}V)$$

Efficient attention is realized by normalizing the query and key using normalization functions $\rho_{q}$ and $\rho_{k}$, respectively, to yield an equivalent dot-product attention output. Studies have demonstrated that these functions are softmax normalization functions. As a result, the method first normalizes the key and query, multiplies the key and value, and then multiplies the resultant global context vector with the query to generate a new representation.

Effective attention is determined not by calculating the similarity between two points, but by employing the attention map $K_{j}^{T}$, where j refers to position j in the input feature, to represent the key with a dimension of $d_{k}$. The derived global attention maps encompass the semantic characteristics of the entire input feature, as opposed to just the similarity with input positions. This approach can significantly reduce computational complexity while maintaining high-level representativeness.

In this study, we introduce the design of the SCA module. The SCA module is devised as a skip connection path to ensure the model’s reusability and augment its localization capacity. The SCA module is integrated into the skip connections, providing each decoder with spatial information to restore intricate details while generating output masks. SCA employs efficient attention but distinguishes between the input features utilized as keys, queries, and values. The output of the skipped encoder layer, denoted as $X_{2}$, is used for querying. The results of the lower decoder layer $X_{1}$ serve as the inputs for keys and values. A linear layer is employed to fuse these two features and scale $X_{1}$ to the same embedding dimension as $X_{2}$. The reason for employing $X_{2}$ as the query input is to formulate a multilevel representation within an effective attention block.

(21) $$X_{1}^{\prime }=FC\left(X_{1}\right),K,V=\mathrm{Proj}\left(X_{1}^{\prime }\right),Q=\mathrm{Proj}\left(X_{2}\right)$$

(22) $$E=\rho_{v}\left(V\right)\rho_{k}\left(K^{T}\right)Q$$

In this paragraph, Q,K,V represent the keys, queries, and values of $X_{1}$ and $X_{2}$, respectively. $\rho_{v}$ and $\rho_{k}$ are normalization functions, Proj is a projection function, which is linear in this instance. E denotes the ultimate output of the SCA module. $X_{1}$ from the encoder is projected linearly and scaled to $X_{1}^{\prime }$, which is then divided into value and key injection. Meanwhile, $X_{2}$ from the decoder is injected into the query, combined with the key to form a context vector, and the output E is produced by merging it with the value.

In preceding sections, we tackled the challenge of noise interference in medical image segmentation and introduced a specialized solution. Subsequently, we developed a model aimed at enhancing the segmentation performance of osseous neoplasm pathology slides, focusing on increasing both accuracy and precision. Our results demonstrate that the proposed model excels in addressing the complications posed by noisy images and yields superior segmentation outcomes compared to existing methods. This study marks a substantial advancement in the field of medical image processing, particularly for osseous neoplasm pathology slides in developing countries. The implications of this research are far-reaching, promising to significantly impact the diagnosis and treatment of osseous neoplasms on a broader scale.

## 4. Simulation Analysis

### 4.1. Experiment Details

The dataset for this study was provided by the Second People’s Hospital of Huaihua and includes high-resolution histological slides of osteosarcoma (OS), stained using the hematoxylin and eosin (H&E) method. These slides were scanned at 40× magnification using the Aperio AT2 Digital Pathology Scanner by Leica Biosystems. A total of 1000 pathology images were initially compiled, from which random areas were captured to generate 10 sub-images per pathology image, each of size 512 × 512 pixels, leading to an aggregate of 10,000 sub-images. After screening for quality and relevance, 2164 sub-images were deemed suitable for training purposes. These images were drawn from the medical records of 284 osteosarcoma patients diagnosed at the hospital between August 2013 and November 2020. Among these patients, 204 met this study’s inclusion criteria.

The demographic and pathological characteristics of the included cases are as follows: the age of the patients ranged from 6 to 82 years, with a mean of 20.05 ± 10.26 years. Notably, 179 patients (87.7%) were under the age of 25. The cohort included 112 males, constituting 54.9% of the study sample. High-grade OS was observed in 163 cases (79.9%), and 138 cases (67.7%) involved tumors located in the extremities. All data were collected from a single hospital, but the medical records indicate that the patients come from various regions across China. To enhance the model’s robustness, geometric transformations like translation were applied to the initial training images. The dataset was further divided into 402 osseous neoplasm pathological sections, with 80% (323 images) used for training the model, and the remaining 20% (comprising 79 images) serving as the validation set to evaluate the model’s performance.

Our experiments were conducted on a computing system with limited performance capabilities. The server operates on an Ubuntu 18.04 LTS environment, powered by an AMD EPYC 7642 central processing unit (CPU) featuring 15 cores running at a clock speed of 2.60 GHz, along with 80 GB of system memory. For graphic-intensive tasks, we employed an NVIDIA RTX 3090 graphics processing unit (GPU), boasting 24 GB of memory, 35.58 TFLOPS of single-precision floating-point computational power, and 71 tensor TFLOPS for half-precision calculations. To optimize GPU-accelerated tasks, the server is also equipped with CUDA version 11.2.

On the software side, our model was developed and trained using PyTorch version 1.8. In addition, we used NumPy 1.19 and Pandas 1.2 for data preprocessing. All coding and testing were conducted in a Jupyter Notebook environment, and version control was maintained through Git, allowing us to keep track of code modifications and experimental versions. Given the constrained hardware resources, we implemented several optimization techniques such as model weight pruning to minimize computational overhead. Additionally, for precise runtime performance monitoring and data visualization, we utilized Grafana and Prometheus. Collectively, this intricate arrangement of hardware and software components is pivotal in achieving efficient and high-speed computations for our machine learning model. This detailed overview offers comprehensive insights into our system’s hardware configurations, software stack, and the optimization techniques employed to circumvent hardware limitations. Such information is invaluable for readers, especially those who might be interested in replicating or improving upon our work.

To bolster the model’s resilience, we applied geometric transformations (e.g., translation) to the initial data images prior to executing the segmentation procedure. We amassed a total of 402 osseous neoplasm pathological sections. Of these, 80% (323 images) were employed for model training, while the remaining 20% (comprising 79 images) was utilized to assess the effectiveness of the fully developed model.

### 4.2. Assessment Measures

To assess the noise suppression effectiveness of our model, we employed the PSNR (peak signal-to-noise ratio) method to examine its denoising proficiency and the resulting image quality. It is commonly defined simply by mean squared error (MSE) as follows:

(23) $$MSE=\frac{1}{mm}\sum_{i=0}^{m-1}\cdot \sum_{j=0}^{n-1}[I(i,j)-K(i,j){]}^{2}$$

In this context, I represents the original image without noise, while K denotes the estimated noise value for I in two m×n grayscale images.

The PSNR, or peak signal-to-noise ratio, is characterized as:

(24) $$PSNR=10\cdot \log_{10}\left(\frac{MAX_{I}^{2}}{MSE}\right)=20\cdot \log_{10}\left(\frac{MAX_{I}}{\sqrt{MSE}}\right)$$

where MAX_I denotes the peak numerical value of the image point color, which is 255 when each sampling point is depicted with 8 bits (e.g., image processing).

Considering that our dataset consists of color images with three RGB values at each point, we redefined the PSNR peak signal-to-noise ratio of color images as follows:

(25) $$PSNR=10\cdot \log_{10}\left(\frac{MAX_{I}^{2}}{\frac{1}{3mn}\sum_{R,G,B}\cdot \sum_{i=0}^{m-1}\cdot \sum_{j=0}^{n-1}\cdot {\left[I_{color}\left(i,j\right)-K_{color}\left(i,j\right)\right]}^{2}}\right)$$

Here,MAX_I still denotes the peak numerical value of the image point color, and RGB represents the color composition. Since there are three color channels, the MSE needs to be divided by 3.

To assess our model’s effectiveness, we employed a blend of metrics like accuracy, IoU, recall, DSC, and F1 score. Moreover, we utilized metrics like true positive (TP), true negative (TN), false positive (FP), and false negative (FN) to comprehend the model’s capability to correctly classify regions as pathological cell nuclei or other regions excluding nuclei. In particular, TP represents regions correctly identified as pathological cell nuclei, TN represents regions correctly identified as other regions excluding nuclei, FP represents regions incorrectly identified as pathological cell nuclei, and FN represents regions incorrectly identified as other regions excluding nuclei [59,60].

Accuracy can be described as:

(26) $$Acc=\frac{TP+TN}{TP+TN+FP+FN}$$

Recall denotes the sensitivity of the model and is defined as:

(27) $$Re=\frac{TP}{TP+FN}$$

IOU (intersection over union) quantifies the resemblance between the anticipated segmentation outcome and the ground truth. We introduce $I_{1}$ as the ground truth region and $I_{2}$ as the predicted region:

(28) $$IoU=\frac{I_{1}\cap I_{2}}{I_{1}\cup I_{2}}$$

DSC (Dice similarity coefficient) serves as a metric of set resemblance frequently employed to determine the likeness between a pair of samples:

(29) $$DSC=\frac{2\cdot \left|I_{1}\cap I_{2}\right|}{\left|I_{1}\right|+\left|I_{2}\right|}$$

Precision refers to the ratio of accurately segmented nucleus and background pixels relative to the overall pixel count in the image:

(30) $$Pre=\frac{TP}{TP+FP}$$

F1-score comprehensively considers both precision and recall, and represents an all-encompassing evaluation metric for both precision and recall:

(31) $$F1=\frac{2\cdot Pre\cdot Re}{Pre+Re}$$

Additionally, we introduce the metric “params” to quantify the total number of parameters in the model. By evaluating both flops and params, we can indirectly gauge the complexity of our algorithmic model. A high parameter count typically implies greater complexity, increased demands on hardware and computational environment, and elevated resource consumption. Recognizing the need to make this technology accessible in developing countries, we made strategic adjustments. While maintaining the accuracy of the model’s results, we streamlined the model architecture and judiciously reduced the number of parameters. This not only lowers the cost of implementing AI-assisted medical systems but also facilitates their broader adoption in resource-limited settings.

### 4.3. Training Strategy

Our training method was to set the epoch to 500 rounds in order to improve the model’s fitting degree and prevent overfitting. At the same time, we monitored the loss and accuracy in real time during the training process. When their trends converged, the training rounds were completed. We used the optimizer SGD and set the batch size to 4 during training to reduce calculation errors. In addition, this method was implemented based on the PyTorch library, a base learning rate of 0.05, weight decay established at 0.0001, and momentum value of 0.9, and an RTX3090 graphics card configuration was employed. We used cross-entropy and Dice loss:

(32) Loss=0.6×Dice+0.4×BCE

### 4.4. Results

**Figure 4:**
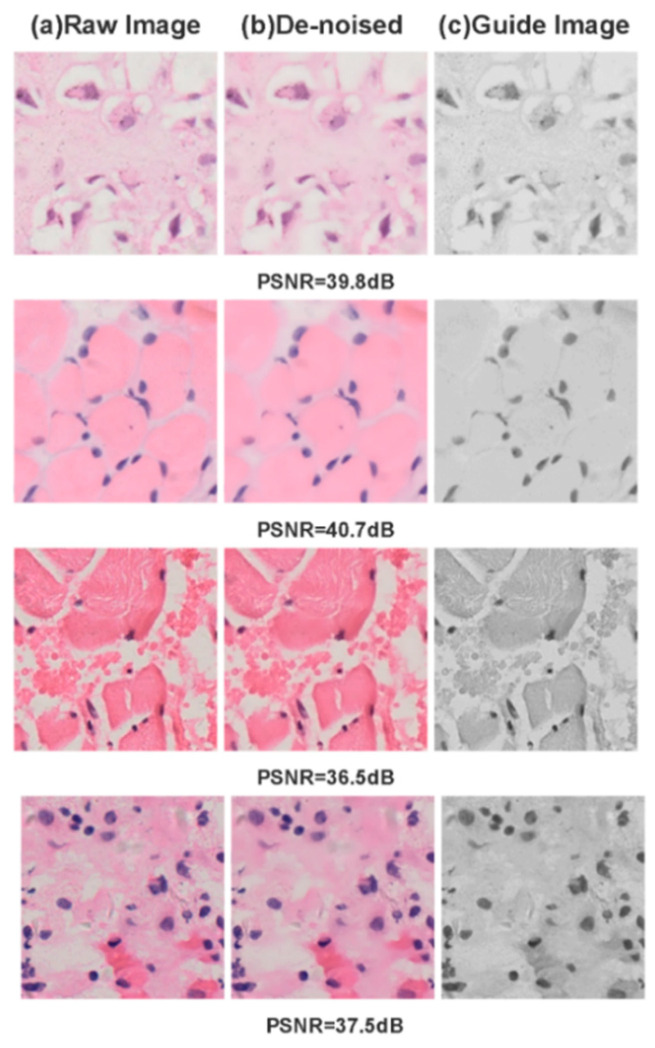
(**a**) Original noisy images; (**b**) images post noise reduction; (**c**) directed grayscale image.

As illustrated in Figure 4, a side-by-side comparison with the original image reveals that our denoised version boasts a markedly improved signal-to-noise ratio. Visually, the denoised image is noticeably crisper and free from noise or interference, a clarity achieved through the guidance of the grayscale image. Upon evaluating various tissue cells, we discovered that most of the denoised images achieved peak signal-to-noise ratio (PSNR) values exceeding 36 dB, with some even surpassing 40 dB—a threshold we consider indicative of excellent denoising performance. Our in-depth analysis shows that the denoising process not only eliminates the majority of surface interference and noise but also accentuates key image features and maintains edge integrity. This enhancement significantly contributes to providing high-quality image inputs for subsequent segmentation models.

**Figure 5:**
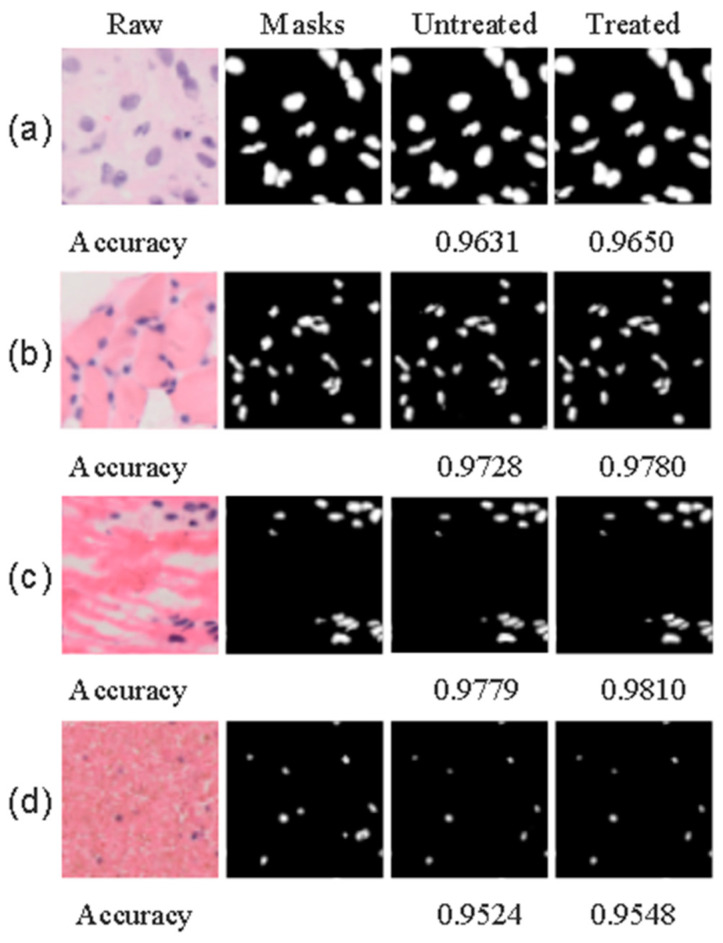
Comparison of prediction image accuracy between denoised and non-denoised processing, (**a**–**d**) represents four different forms of osteosarcoma cells with different pathologies.

Figure 5 shows the predicted images obtained by directly segmenting the images without directed filtering denoising, and the images processed using denoising and then segmentation and prediction. The results indicate that directed filtering can effectively eliminate a substantial quantity of noisy data, and ultimately enhance the precision of the model segmentation.

Pathological section images of osseous neoplasms are composed of two main elements: cell nuclei and surrounding areas. Our primary objective is to precisely segment the cell nuclei from the rest of the osseous neoplasm tissue. To assess the effectiveness of our approach, we conducted a comparative analysis with a baseline model to demonstrate its superior performance.

**Figure 6:**
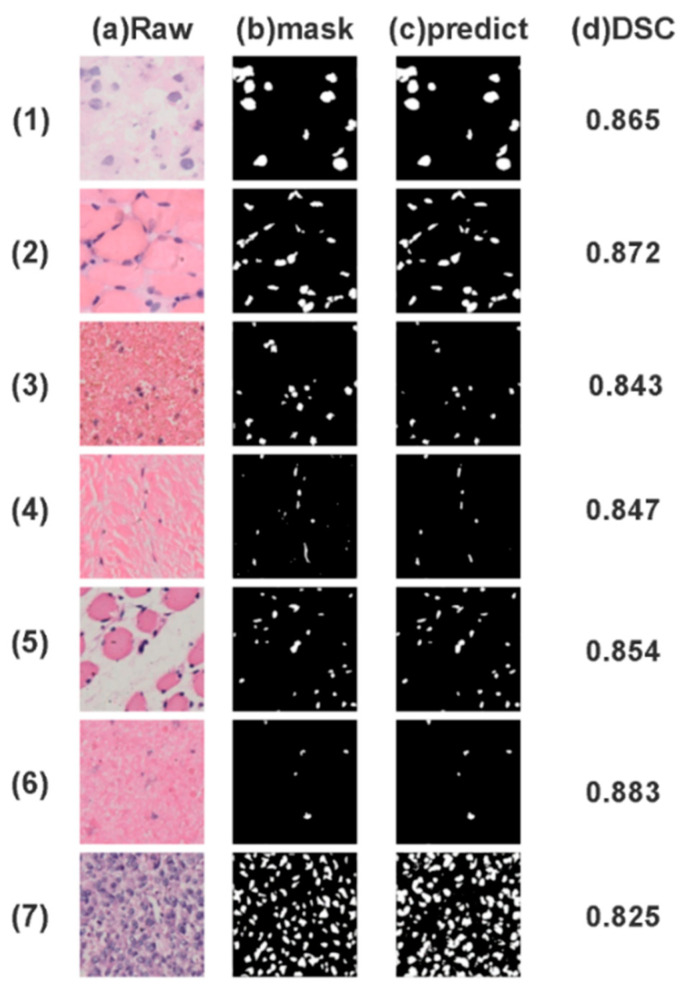
Representative of prediction results of different tissues and cells of osseous neoplasm. (1)–(7) are the display of the original images, marker recognition, and the predicted results of our model in various pathological sections of osteosarcoma with different characteristics.

In Figure 6, subfigure (a) displays the original pathological slice, while (b) illustrates the manually labeled mask, and (c) presents the model’s final prediction. Given that pathological slices of osseous neoplasm tissues often encapsulate a vast amount of data within a single image—sometimes containing over a hundred cell nuclei to be labeled—the model is faced with stringent requirements for precise identification and labeling of these nuclei. Our approach demonstrates high-performance capabilities across a range of tissue characteristics and cell types. Through visual comparison between our model’s predicted results and manually labeled samples, it is evident that our methodology provides healthcare professionals with a reliable tool for the accurate identification of cell nuclei within osseous neoplasm tissues.

**Figure 7:**
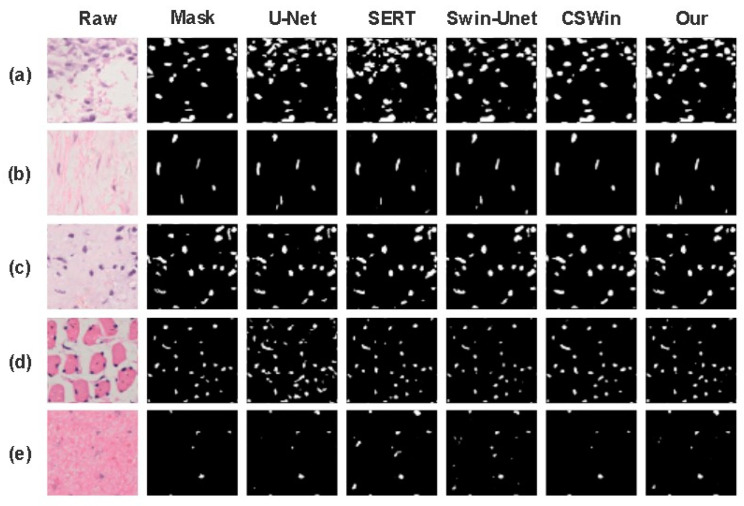
Comparing the segmentation results of various models, where (**a**–**e**) denote the predicted images of distinct osseous neoplasm tissues and cells post-segmentation.

From Figure 7, we can examine the segmentation outcomes from various models on cell histopathological slices. There is the pathological image followed by the label image; U-Net; SERT; Swin-Unet; Cswin-Transformer; and our model’s segmentation rendering. We can visually see the segmentation differences among these models. Additionally, we can see from the figure that the distinction between our model and the ground truth mask is comparatively smaller compared to the other models. The accuracy of our model can be intuitively seen in the five different tissue cell pathology sections (a) to (e), especially in the last tissue cell section (e), where our model demonstrates excellent detection results for small nucleus regions.

In Table 2, we juxtapose our model against other models, including U-Net [47], U-Net++ [61], SERT [62], and CSwin-transformer [63], based on various evaluation metrics. The assessment outcomes suggest that our model generally surpasses the other models in terms of recall (Re), precision (Pre), etc., our model demonstrates a significant edge in DSC, which is 1.4% higher than the second best model [64]. Despite the fact that our method contains a greater quantity of parameters in comparison to U-Net, its FLOPs count is less than that of most convolutional and non-convolutional models. This provides a computational foundation for our model’s application in developing countries, mitigating the high computational cost. Furthermore, our model is optimized with noise reduction techniques, leading to enhancements in accuracy, DSC, and F1-score.

**Figure 8:**
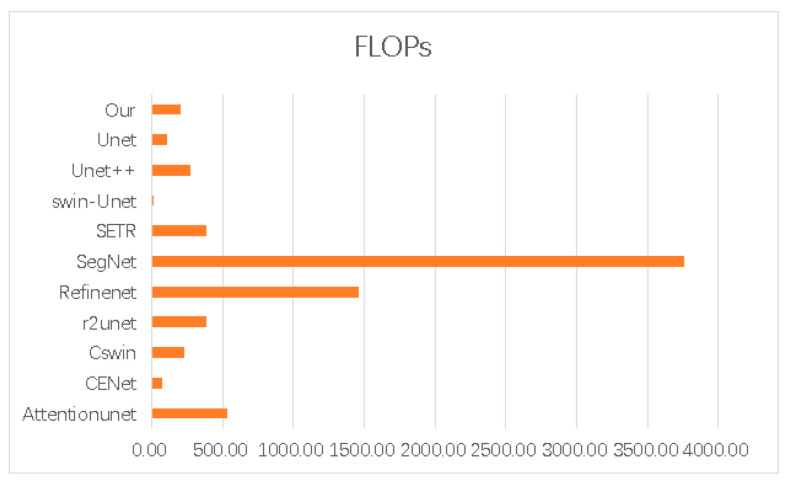
Comparing the FLOPs (floating-point operations per second) of various segmentation models.

**Figure 9:**
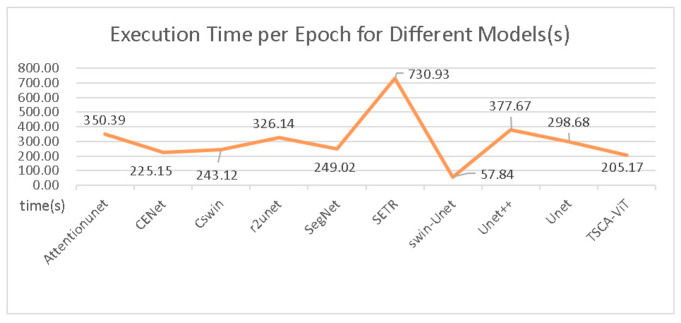
Comparison of epoch runtime for different models under the same GPU.

Figure 8 and Figure 9 provide an insightful comparison of computational performance across various models. Our bar charts clearly show that our model outperforms its competitors in terms of computational efficiency. Figure 8 features a total of 11 models and displays the floating-point operations per second (FLOPs) for each. Among these models, Attention-UNet exhibits the highest number of FLOPs, exceeding 500. In contrast, our model exhibits a markedly lower level of computational complexity. This advantage is further highlighted in Figure 9, which reveals the time each model takes to complete a single epoch under identical GPU conditions. Our model distinguishes itself by finishing an epoch in just 205.17 s—significantly faster than any other model tested. In summary, our model excels in balancing high accuracy and efficient attention mechanisms with reduced time complexity. This efficiency allows our model to complete epochs more quickly than its peers, making it particularly advantageous for real-world applications.

**Figure 10:**
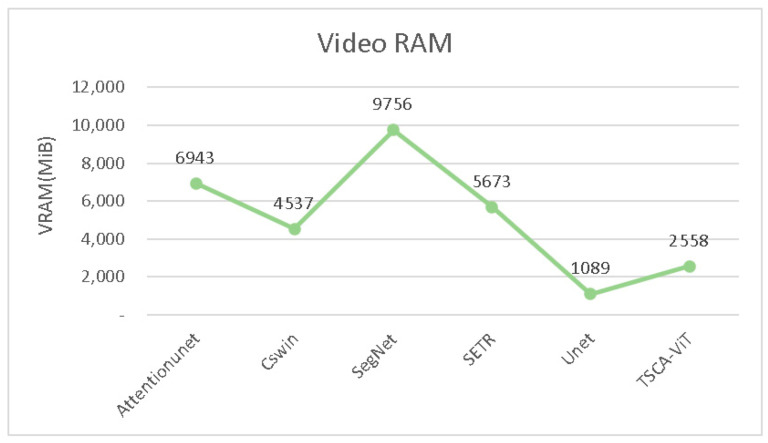
Comparative analysis of training time VRAM usage for traditional convolutional models and their improved versions on a single RTX 3090 GPU.

In Figure 10, we conduct a comparative analysis that examines the training time VRAM consumption between our proposed model and established image segmentation models like U-Net. All measurements were obtained using a single RTX 3090 graphics card and captured through NVIDIA’s nvidia-smi tool. Our findings reveal that while our model does consume slightly more VRAM than the traditional U-Net convolutional model, it is considerably more efficient in terms of memory usage compared to other enhanced segmentation models.

This finding demonstrates the efficiency of our model, capable of achieving optimal image segmentation results using less memory resources. This effectively strikes a balance between handling complex image segmentation tasks and minimizing hardware resource demands. Therefore, despite slightly exceeding U-Net in terms of VRAM usage, our model still exhibits remarkable advantages in VRAM utilization efficiency, especially when compared to other advanced models.

**Figure 11:**
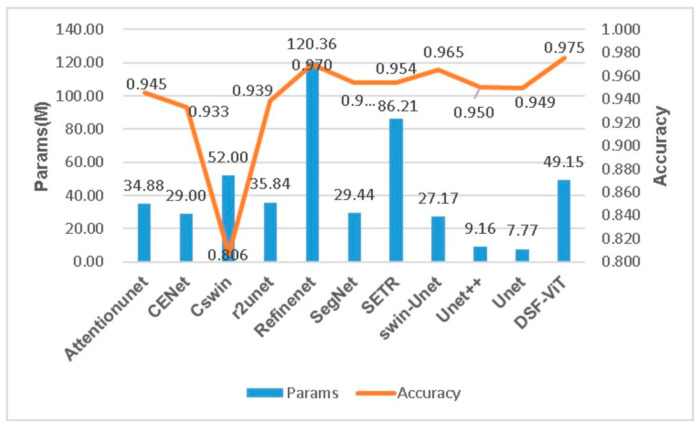
Examining the accuracy of diverse segmentation models with varying parameter configurations.

**Figure 12:**
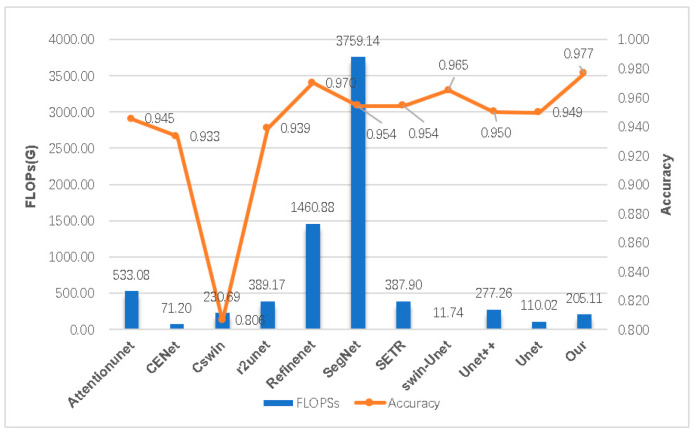
Comparing the accuracy of various segmentation models across distinct FLOP (floating-point operations per second) levels.

Figure 11 and Figure 12 display the performance of accuracy under varying parameter configurations and different FLOPs, respectively. These figures illustrate that our model achieves significantly higher accuracy than other models at comparable time complexities. Certain models, such as RefineNet, possess a FLOP calculation amount within their parameters that is over seven times higher than ours, even though their accuracies are marginally lower. In comparison, our model excels in performance by minimizing complexity while ensuring the highest accuracy in both time and space complexity.

**Figure 13:**
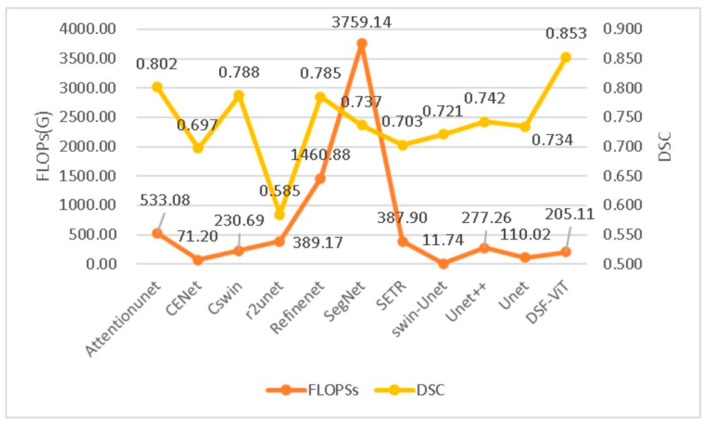
Evaluating DSC (Dice similarity coefficient) performance across various models.

Figure 13 compares the DSC among various models. The chart reveals that our model not only has the highest DSC, suggesting better stability than other models, but also requires a lower FLOP calculation amount compared to most competing models. Overall, our model maintains stability with the least calculation amount.

**Figure 14:**
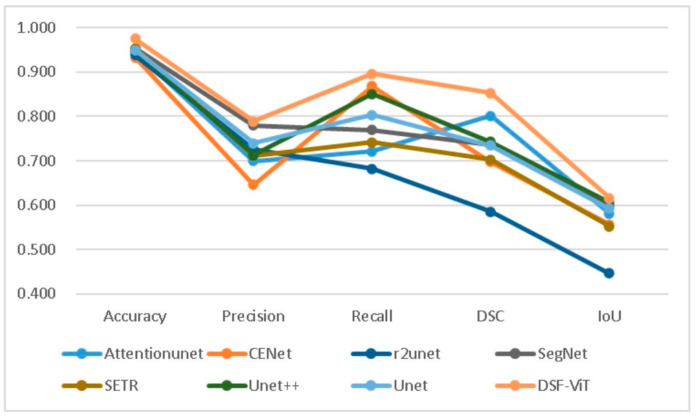
Assessing the performance of diverse segmentation models in comparison.

**Figure 15:**
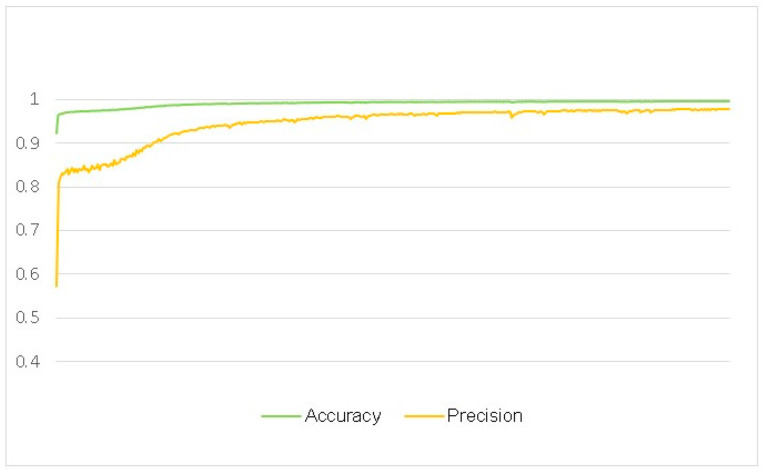
Changes in precision and accuracy during training.

Figure 14 compares different performance indicators across all models. It is clear that our model outperforms similar models with respect to recall, accuracy, and stability metrics. Figure 15 showcases some changes during the training process of precision and accuracy, proving that our model has high accuracy and precision. It can truly assist doctors in making scientific and professional judgments on diagnostic results.

### 4.5. Discussion

In this study, we engineered a highly efficient and robust AI-driven medical support system aimed at the semantic segmentation of cell nuclei in pathological slides of osseous neoplasms. This system holds significant promise for augmenting medical care and expediting the diagnosis of such conditions, particularly in developing countries—adding tangible, real-world clinical value. Our model, enhanced using advanced noise reduction techniques, adeptly processes clinical pathology slides to deliver highly accurate cell nucleus segmentation outcomes.

Our adoption of a directed filtering approach, which leverages the grayscale information of the original image, yielded significant noise elimination benefits, thereby augmenting the model’s overall accuracy. At the core of our research is the segmentation model, which utilizes a pioneering pure Transformer architecture that forgoes convolution in favor of dual attention mechanisms. These techniques skillfully capture relationships across all feature dimensions—both spatial and channel-wise—while maintaining computational efficiency. By incorporating a skip path equipped with a cross-attention module, we further elevated our model’s localization prowess.

Our model stands out for its computational efficiency and small parameter size, traits particularly valuable in the medical environments of developing countries. Compared to most convolutional models of the same accuracy level, our model expedites patient condition assessments by reducing computation time significantly. It demonstrates robust and efficient performance across different tissue cell datasets of osseous neoplasm pathology slides, thus providing significant assistance to doctors, improving their work efficiency and speeding up clinical diagnoses.

While our model shows promise, there are several limitations and sources of uncertainty that warrant further attention. Firstly, the dataset primarily originates from the Second People’s Hospital of Huaihua, potentially limiting its broader applicability. While the 1000 pathological slides from the Second People’s Hospital of Huaihua provided us with valuable data, the volume remains limited compared to larger, multicenter datasets. This might impact the diversity of disease characteristics we are able to capture. Due to the smaller size of our dataset, there is potential for our model to overfit or possibly miss out on more intricate patterns. Consequently, our findings might be influenced by this limitation. Secondly, the model’s interpretability is low, potentially making it less trustworthy for nonexperts. Thirdly, our approach relies heavily on manually annotated data, adding to the workload and introducing potential bias. Fourthly, the model is tailored specifically for osteosarcoma pathological slides, and its applicability to other contexts such as MRI semantic segmentation remains questionable, particularly in under-resourced medical environments in developing countries. Lastly, it is crucial to note that the practical implementation of this AI system in a clinical setting presents its own set of challenges, including data privacy concerns, integration with existing healthcare systems, and the need for extensive validation and testing to meet clinical standards.

Various forms of uncertainty also contribute to the limitations of our study. Hyperparameter uncertainty, such as the choice of learning rate and regularization terms, can impact predictive reliability. Model parameter uncertainty arises from the initialization and optimization pathways employed during training, potentially affecting the model’s stability and predictive capability. Dataset uncertainties, including noise, outliers, or missing values, can adversely affect prediction accuracy. Lastly, the one-time training–test split introduces uncertainty, as model performance could vary due to the randomness of this division.

These limitations and uncertainties indicate avenues for future research and improvement, including diversification of datasets, enhanced model interpretability, and more robust statistical validation methods.

Our future research endeavors are geared towards addressing the existing limitations of our diagnostic model, with plans for both methodological and clinical enhancements. In collaboration with the Second People’s Hospital of Huaihua, we aim to gather more detailed pathological data from osteosarcoma patients to optimize our model further. Specific evaluation metrics may include segmentation accuracy, improvements in physician workflow efficiency, and increased patient diagnostic accuracy. A preliminary clinical trial is planned in partnership with the orthopedic department of the hospital, aiming to recruit at least 20 osteosarcoma patients and 5 specialized physicians. This trial will leverage our model for cellular nucleus segmentation and compare its performance against traditional methods. Moreover, as part of the trial, we will introduce a patient tracking mechanism that will enable comparative evaluations across different stages of a patient’s illness. Prognosis assessment will also be integrated into our approach. This layered analysis will further serve as a basis for comparisons between evaluations conducted by multiple physicians on the same patient, thereby enhancing the model’s clinical utility.

Once again, for artificial intelligence systems, the method we propose can only serve an auxiliary role and as a reference for doctors’ diagnoses. We expect to complete this trial within the next 12 months. Furthermore, statistical tests such as t-tests or ANOVA will be used to evaluate statistically significant differences in predictive performance between our model and conventional methods in aspects like segmentation accuracy, physician workflow efficiency, and patient diagnostic accuracy.

Concurrently, we will work on diversifying clinical datasets, implementing advanced denoising techniques, and exploring more efficient attention mechanisms to reduce computational costs. We believe that these efforts collectively represent a significant step toward enabling more efficient and accurate diagnosis and treatment procedures, particularly in resource-limited settings. The results of our ongoing and future work indicate promising directions for continued research in this field.

## 5. Conclusions

In this study, we unveiled a comprehensive methodology for the semantic segmentation of cell nuclei in pathological sections of osseous neoplasms. Our approach began with an efficient and swift noise reduction process tailored for clinically sourced pathological images. This was followed by the deployment of a groundbreaking pure Transformer network architecture, which features an innovative twin attention mechanism. This design not only reduces the model’s computational burden but also offers a pioneering avenue for processing pathological analyses in resource-constrained settings, such as developing countries. Our empirical results affirm that our methodology excels in both accuracy and efficiency, while markedly reducing computational complexity when compared to traditional neural network architectures.

As we look to the future, we are planning to integrate preprocessing modules like image screening to filter out a substantial volume of potentially irrelevant image data from pathological sections. By weaving in artificial intelligence technologies, we aim to extend our algorithm to encompass visual characteristics, including image texture, into our segmentation evaluation framework. When integrated with clinical diagnostic outcomes, this approach promises to further streamline data processing, heightening both the precision and efficacy of our model.

## Figures and Tables

**Table 1:** Symbol description.

Symbol	Paraphrase
E	Efficient attention
G	Global context vectors
Q	Query
$n_{1}$	One parameter of $X_{1}$
$n_{2}$	$n_{1}$ after linear conversion
$\rho_{K}(K{)}^{T}$	Normalization functions for the keys
$\begin{array}{c} \rho v(V) \end{array}$	Normalization functions for the values
d	d is the embedding dimension
$d_{q}$	The embedding dimension of queries
$d_{k}$	The embedding dimension of keys
$d_{v}$	The embedding dimension of values
$X_{1}$	Output of the decoder layer
$X_{2}$	Output of the encoder layer
$X_{1}^{\prime }$	$X_{1}\;$ after linear conversion

**Table 2:** Evaluating diverse pathological section datasets.

Model	Ac	Pr	Re	DSC	F1	IoU	Params	FOLPs	AUC
Attention-Unet	0.945	0.699	0.721	0.801	0.710	0.581	34.88 M	533.08 G	0.897
CENet	0.933	0.646	0.868	0.696	0.691	0.556	29.53 M	71.2 G	0.893
CSwin-transfomer	0.806	0.802	0.806	0.788	0.793	0.710	52.15 M	230.69 G	0.886
R2U-Net	0.938	0.726	0.682	0.584	0.703	0.446	35.84 M	389.17 G	0.907
SegNet	0.954	0.780	0.769	0.736	0.774	0.598	29.44 M	3759.14 G	0.887
SERT	0.954	0.712	0.741	0.702	0.726	0.551	86.21 M	387.9 G	0.945
Swin-Unet	0.965	0.614	0.913	0.721	0.734	0.571	27.17 M	11.74 G	0.941
UNet++	0.951	0.713	0.851	0.742	0.776	0.605	9.16 M	277.26 G	0.910
U-Net	0.955	0.740	0.803	0.734	0.770	0.592	7.77 M	110.02 G	0.878
Our (TSCA-ViT)	0.975	0.789	0.896	0.853	0.832	0.616	49.15 M	205.11 G	0.952
Our (denoise+TSCA-ViT)	0.977	0.803	0.893	0.855	0.834	0.619	49.15 M	210.23 G	0.952

## Data Availability

Data used to support the findings of this study are currently under embargo while the research findings are commercialized. Requests for data, 12 months after publication of this article, will be considered by the corresponding author.
